# On Consensus and Stability under Denial-of-Service Attacks

**DOI:** 10.3390/e24020154

**Published:** 2022-01-20

**Authors:** Ewa Girejko

**Affiliations:** Faculty of Computer Science, Bialystok University of Technology, 15-351 Białystok, Poland; e.girejko@pb.edu.pl

**Keywords:** multi-agent system, switched systems, discrete systems, DoS attacks, exponential stability, consensus

## Abstract

In the paper, discrete-time multi-agent systems under Denial-of-Service (DoS) attacks are considered. Since in the presence of DoS attacks the stability of the whole system may be disturbed, sufficient stability conditions for the multi-agent system under DoS attacks are delivered. The consensus problem for the special case of the considered system under DoS attacks is also examined by delivering sufficient conditions. Theoretical considerations are illustrated by numerical examples.

## 1. Introduction

Nowadays, cyber attacks on networks of cooperating devices are one of the most troublesome threats that disrupt or interrupt entire systems. A priority is to ensure the security of industrial control systems based on the flow of information and communication technologies. A major concern is that cyber-attackers may be able to break connections in control systems that are utilized in power grids, transportation, food distribution, and many other services important to society. Therefore, it is critical to assess and improve the security of such control systems and ensure resilience against cyber attacks. This will result in protecting the environment against financial losses and other possible damages. Motivated by the above, an enormous number of researchers have been attracted to work on these kind of problems; see for example [1,2,3,4,5,6,7].

In general, in the literature, the problem of how Denial-of-Service (DoS) attacks interrupt entire systems is explored from the point of view of feedback control, state estimation, and multi-agent consensus problems, and one can find different approaches to solutions of these problems. A very interesting approach focuses on multi-agent consensus problems under DoS attacks [8,9,10,11,12,13]. In the mentioned papers, authors characterize the communication topology of multi-agent systems with an undirected graph represented by nodes (agents) and edges (communication links).

In [8,9,10], researchers proposed control law and interaction rules to ensure consensus under DoS attacks. They considered the case in which the jamming attacker can target all communication links at once. In particular, Senejohnny et al. [8] used a self-triggering approach: when a triggering condition holds, each agent attempts to communicate.

In the modified problem formulation in [9], multiple jamming intruders attack individual communications links.

The works discussed above concern scalar dynamics, while in [11], the authors explore the same problem with multi-dimensional dynamics. In addition, an apparently different game-theoretical formulation of multi-agent systems under DoS attacks that target individual links is presented in [12,13].

Particularly interesting is the problem of how Denial-of-Service attacks disrupt the exponential stability of systems, which is investigated in, for example [1,14,15]; see also the references therein.

Following this lead, we decided to investigate the stability problem of systems under DoS attacks. In the first work devoted to this problem (see [16]), we examined stability in the presence of DoS attacks of multi-agent systems (MAS) defined on time scales. However, there is an enormous number of scientists who study such problems for systems with discrete time and only a narrow group of researchers who deal with similar problems on time scales. Moreover, this subject is also closely related to the problem of consensus with a leader for multi-agent systems. Taking all this into account, we decided to study behaviour (stability and leader-following consensus) of the discrete multi-agent systems. Since in the presence of DoS attacks, the information flow between devices can be interrupted, which in mathematical language means that stability of the whole system is disturbed, we propose sufficient conditions for the stability of the MAS. To tackle this problem, the distributed control law guaranteeing stability of the system dynamics despite DoS attacks is delivered. In order to improve the resilience of the network, we propose a control technique that modifies the coupling strength parameter after an attack on the system. In our previous paper, we examined only the stability of the system, paying no attention to the consensus problem. In this work, we cope with the leader-following consensus problem of the multi-agent system in the presence of DoS attacks by employing switched symmetric error systems and proving their exponential stability under arbitrary switching in base-of-Schur stability. This results in the formulation of sufficient conditions for the multi-agent system to achieve a consensus under DoS attacks. The main contribution of this paper can be described in terms of three aspects:

1. Discrete-time multi-agent systems under Denial-of-Service (DoS) attacks are investigated in terms of the leader-consensus problem in a DoS attacks situation, and sufficient conditions ensuring such a consensus are delivered;
2. Stability protocol under DoS attacks on the considered systems is proposed in order to guarantee stability of the system in the presence of DoS attacks;
3. Numerical analysis of the theoretical investigation is given to illustrate presented results.

We organize the paper as follows. In Section 2, the preliminaries from the graph theory are given, while in Section 3, we formulate the statement of the problem of behaviour of systems under DoS attacks. Further, in Section 4, we derive sufficient conditions guaranteeing exponential stability of the system under DoS attacks. Consensus problem analysis is given in Section 5. To be more precise, we deliver conditions under which the consensus is achieved in the multi-agent system with a leader in spite of DoS attacks. Illustrative examples are presented to verify the theoretical consideration. Finally, we conclude the paper in the last section.

## 2. Preliminaries

We start with some notions from graph theory. By G=(V,E) we denote a weighted communication graph of *n* agents, by $V=\{v_{1},v_{2},\ldots ,v_{n}\}$ the set of nodes (vertices), and by E⊆V×V the set of edges. If information flows from agent *j* to agent *i*, then we denote it as edge (i,j). Entries of the adjacency matrix $A=\left[a_{ij}\right]\in R^{n\times n}$ are defined by $a_{ij}=1$ if (i,j)∈E, and $a_{ij}=0$ if (i,j)∉E. Matrix $L=\left[l_{ij}\right]\in R^{n\times n}$ is called a Laplacian matrix induced by the topology *G* if $l_{ii}=\sum_{i\neq j}a_{ij}$ and $l_{ij}=-a_{ij}$, i≠j, where $a_{ij}$ are the entries of the adjacency matrix *A*. We observe that there exists at least one zero eigenvalue of matrix *L* with a corresponding eigenvector $1_{n}={\left[1,\ldots ,1\right]}^{T}$. Graph *G* is called undirected if for every (i,j)∈E we have (j,i)∈E. It is easy to see that matrices *A* and *L* are symmetric for any undirected graph, and we get $0=\lambda_{1}\leq \lambda_{2}\leq \ldots \leq \lambda_{n}$ for $\lambda_{i}$, i=1,…,n with eigenvalues of *L*. Let us also recall that if there exists an edge between any two different vertices, then an undirected graph is connected. Moreover, we get $\lambda_{2}>0$ if the graph is connected.

Throughout the paper, all graphs are assumed to be finite, undirected, and without loops or multiple edges.

In the proposed model of MAS under DoS attacks, we employ discrete-time switched linear systems.

A discrete-time switched linear system under arbitrary switching is an inclusion of the following form,

(1) $$x\left(t+1\right)\in {\left\{M_{\kappa }x\left(t\right)\right\}}_{\kappa \in I}\;,\;\;x\left(0\right)=x_{0}\;,$$

where $x\left(t\right)\in R^{n}$ is the state vector, x(0) is initial condition, $M_{\kappa }\in R^{n\times n}$, and *I* is a finite index set. The switched system with a specific switching pattern is denoted by

$$x\left(t+1\right)=M_{\kappa (t)}x\left(t\right)\;,\;\;x\left(0\right)=x_{0}\;,$$

where $\kappa :Z_{+}\to I$ is a piecewise continuous switching signal. Here, $Z_{+}$ denotes the set of all nonnegative integers.

**Definition** **1** ([17]). *Switched system (1) is exponentially stable if $∥x\left(t\right)∥\leq \mu^{t}∥x_{0}∥$ with 0<μ<1 holds for any $t\in Z_{+}$ and any initial state $x_{0}$.*

The following theorem will be useful for deriving the main results.

**Theorem** **1** (cf. [17], Theorem 1). *Let ${\left\{M_{\kappa }\right\}}_{\kappa \in I}$ be a family of symmetric matrices. If all matrices in the family ${\left\{M_{\kappa }\right\}}_{\kappa \in I}$ are Schur stable, then switched system (1) is exponentially stable under arbitrary switching.*

## 3. Problem Statement

Consider a multi-agent networked system consisting of *N* agents. The interaction topology of a network is described by undirected graph *G* with the corresponding adjacency matrix $A=\left[a_{ij}\right]\in R^{N\times N}$ and the Laplacian matrix *L*. The neighbours of agent *i* are denoted by $N_{i}=\left\{j\in V|\left(j,i\right)\in E\right\}$. Each node of graph *G* represents a dynamic agent with the dynamics

(2) $$x_{i}\left(t+1\right)=ax_{i}\left(t\right)+bu_{i}\left(t\right)\;,\;t\in Z_{+},\;i=1,\ldots ,N,$$

where $x_{i}\left(t\right)\in R$ and $u_{i}\left(t\right)\in R$ denote the state and the control input at time *t*, respectively. The constant real parameters *a* and *b* (coupling strengths) will be specified later.

In the sequel, we assume that DoS attacks can occur on some or all transmission channels at any time. We define

- $D_{\left(i,j\right)}\left(Z_{+}\right)$, i<j, to be the union of moments of DoS attacks on channel (i,j)∈E over $Z_{+}$;
- $\Gamma \left(t\right):=\{\left(i,j\right)\in E|t\in D_{\left(i,j\right)}\left(Z_{+}\right)\}$ to be the set of channels that are attacked at time *t*.

Since graph *G* is undirected, only edge (i,j) with i<j is considered and $D_{\left(i,j\right)}=D_{\left(j,i\right)}$.

We set Laplacian matrix $L_{\Gamma (t)}$ with entries $l_{ij}=0$ for (j,i)∉Γ(t); that is, if channel (j,i) is not attacked at time *t*. According to the definition, matrix $L_{\Gamma (t)}$ describes DoS attack at time *t*. Next, let us denote by Ω a set of all subsets of the set of all connections between every two different nodes in graph *G*. To be more precise, setting E={(i,j):1≤i,j≤N∧i<j}, we can write that Ω is the set of all subsets of the set E and, what follows, $\left|\Omega \right|=2^{\left|E\right|}$. One can observe that the definition of Γ gives an index for the attack modes. Therefore, for a given t∈N, there are $2^{\frac{\left|E\right|}{2}}=2^{\left|E\right|}$ possible different attack modes. By introducing a bijection map $f:\Omega \to \{1,\ldots ,2^{\left|E\right|}\}\subset N$, we define switching signal $\kappa :Z_{+}\to \left\{1,\ldots ,2^{\left|E\right|}\right\}=I$ as κ(t):=f(Γ(t)), which is piecewise continuous. In this way, every DoS attack mode is described by matrix $A_{\kappa (t)}$ as follows:

(3) $$A_{\kappa (t)}:=L-L_{\Gamma (t)},\;t\in Z_{+}.$$

## 4. Stability Protocol under DoS Attacks

In this section, we present how to design a control protocol that solves the stability problem under DoS attacks.

The state-feedback distributed control for multi-agent system (2) is proposed as follows

(4) $$u_{i}\left(t\right)=\sum_{j\in N_{i},\left(j,i\right)\notin \Gamma \left(t\right)}a_{ij}\left(x_{j}\left(t\right)-x_{i}\left(t\right)\right)\;,\;\;i=1,\ldots ,N\;.$$

Then the collective dynamics of a multi-agent system (2) following protocol (4) can be written in the following matrix form

(5) $$x\left(t+1\right)=\left(aI_{N}-b\left(L-L_{\Gamma (t)}\right)\right)x\left(t\right)\;,\;x\left(0\right)=x_{0}\;,$$

where $x={\left[x_{1},\ldots ,x_{N}\right]}^{T}$, $I_{N}$ is an identity matrix of dimension N×N, and *L* and $L_{\Gamma (t)}$ are the Laplacian matrices of an appropriate dimension. According to Formulas (1) and (3), we obtain the switched system

(6) $$x\left(t+1\right)\in {\left\{\left(aI_{N}-bA_{\kappa }\right)x\left(t\right)\right\}}_{\kappa \in I}\;,\;x\left(0\right)=x_{0}\in R^{N}$$

that represents multi-agent system (5) under DoS attacks. Here, $x_{0}$ denotes the initial state for system (5).

**Theorem** **2.** *If all matrices in the family ${\left\{aI_{N}-bA_{\kappa }\right\}}_{\kappa \in I}$ are Schur stable, then a multi-agent system (5) under DoS attacks is exponentially stable.*

**Proof.**  Observe that multi-agent system (5) under DoS attacks is described by a switched system (6). Since all matrices in the family ${\left\{aI_{N}-bA_{\kappa }\right\}}_{\kappa \in I}$ are symmetric and Schur stable, the claim follows by Theorem 1. □

Let us define the following

$$spec\left(A_{\kappa }\right):=\left\{\lambda_{j}^{\kappa }:j=1,\ldots ,N\right\},\;\;\kappa \in I$$

and

$$\lambda_{max}:=\max_{j\in \{1,\ldots ,N\},\kappa \in I}\lambda_{j}^{\kappa }\;.$$

**Proposition** **1.** *Assume that a∈(−1,1) in system (5). If for b<0 holds $\frac{a-1}{b}>\lambda_{max}$ or for b>0 holds $\frac{1}{b}\left(1+a\right)>\lambda_{max}$; then, a multi-agent system (5) under DoS attacks will be exponentially stable.*

**Proof.**  First let us observe that, due to Theorem 2, if all matrices in the set ${\left\{aI_{N}-bA_{\kappa }\right\}}_{\kappa \in I}$ are Schur stable, then system (5) has the equilibrium x(t)≡0 exponentially stable, in spite of DoS attacks. We notice that $spec{\left\{aI_{N}-bA_{\kappa }\right\}}_{\kappa \in I}=\left\{a-b\lambda_{j}^{\kappa }:j=1,\ldots ,N,\kappa \in I\right\}$. Therefore, we have to show that $|a-b\lambda_{j}^{\kappa }|<1$ for all j=1,…,N and κ∈I. Since $0\in spec(A_{\kappa })$, κ∈I, it follows that a∈(−1,1). We show that first condition, namely that for b<0, $\frac{a-1}{b}>\lambda_{max}$ holds, implies Schur stability of all matrices in ${\left\{aI_{N}-bA_{\kappa }\right\}}_{\kappa \in I}$. Indeed, since b<0, $\frac{a-1}{b}>\lambda_{max}$ and a∈(−1,1), it follows that:

$$a-1-b\lambda_{j}^{\kappa }<0\;\;\mathrm{and}\;a+1-b\lambda_{j}^{\kappa }>0,$$

for all κ∈I,j∈{1,…,N}. The proof for the second case is analogous. □

**Remark** **1.** *Observe that if b=0 in system (5), then it is enough that a∈(−1,1) for system (5) to be exponentially stable.*

## 5. Consensus with a Leader under DoS Attacks

In this section, we investigate multi-agent system (2) with a,b=1 but with a leader. Therefore, the model of *N* agents is described as follows:

(7) $$x_{i}\left(t+1\right)=x_{i}\left(t\right)+u_{i}\left(t\right)\;,\;t\geq 0,\;i=1,2,\ldots ,N\;,$$

while the dynamics of a leader, labelled by *l*, are given by

(8) $$x_{l}\left(t+1\right)=x_{l}\left(t\right)+f\left(t\right)\;,\;t\geq 0,$$

where $f:Z_{+}\to R$. If f(t)≡0, then $x_{l}\left(t\right)\equiv constant$ (constant reference state). In the opposite case, it is time-varying reference state.

**Definition** **2.** *Multi-agent system (7) and (8) is said to achieve a consensus with a leader if*

(9) $$\lim_{t\to \infty }\left|x_{i}\left(t\right)-x_{l}\left(t\right)\right|=0\;,\forall i\in \{1,2,\ldots ,N\}\;$$

*for any initial conditions: $x_{l}\left(0\right)$, $x_{i}\left(0\right)$, i=1,…,N.*

The state-feedback distributed consensus control for agent *i* is expressed as follows:

(10) $$u_{i}\left(t\right)=f\left(t\right)-\beta \left[\sum_{j\in N_{i},\left(j,i\right)\notin \Gamma \left(t\right)}a_{ij}\left(x_{i}\left(t\right)-x_{j}\left(t\right)\right)+b_{i}\left(x_{i}\left(t\right)-x_{l}\left(t\right)\right)\right]\;\;i=1,2,\ldots ,N,$$

where $a_{ij}$ (i,j=1,2,…,N) is the (i,j)th entry of the adjacency matrix $A\in R^{N\times N}$, $b_{i}>0$ if there is information flow from a leader to agent *i* and $b_{i}=0$; otherwise, β>0 is the coupling strength that will be specified later. We assume that not all $b_{i}$’s are equal to zero.

**Remark** **2.** *One can observe that the assumption that there exists i∈{1,2,…,N} such that $b_{i}\neq 0$ means that a leader always has influence on at least one agent. Moreover, since the entries of adjacency matrix are not all zeros at the same time, this implies that there is always information flow between agents, which ensures the leader’s influence is also spread over other agents. Finally, in the case $b_{i}\neq 0$ for all i=1,…,N, we have the strongest leader-dependence situation when all agents are directly influenced by the leader.*

On account of consensus protocol (10), we have

(11) $$x\left(t+1\right)=f\left(t\right)1_{N}+x\left(t\right)-\beta \left(L-L_{\Gamma (t)}+B\right)x\left(t\right)+\beta Bx_{l}\left(t\right)1_{N}\;,$$

where $x={\left[x_{1},\ldots ,x_{N}\right]}^{T}$, $B:=\mathrm{diag}\left\{b_{1},\ldots ,b_{N}\right\}\in R^{N\times N}$ is a diagonal matrix with nonzero trace, $1_{N}$ is a column of N×1, and $I_{N}$ is an N×N-identity matrix. Now, applying Formulas (1) and (3), as in the previous section, we obtain the switched system

(12) $$x\left(t+1\right)\in \left\{f\left(t\right)1_{N}+x\left(t\right)-\beta \left(A_{\kappa }+B\right)x\left(t\right)+\beta Bx_{l}\left(t\right)1_{n}\right\}{\;}_{\kappa \in I}$$

that gathers all possible DoS attacks on system (11). Now let us define an error vector $e\left(t\right)={\left[e_{1}\left(t\right),\cdots ,e_{N}\left(t\right)\right]}^{T}$ with $e_{i}=x_{i}-x_{l}$. Then we get

$$\begin{array}{cc} e(t+1)= & x\left(t+1\right)-x_{l}\left(t+1\right)1_{N}\\ = & f\left(t\right)1_{N}+x\left(t\right)+\left(I_{N}-\beta \left(A_{\kappa (t)}+B\right)\right)x\left(t\right)+\beta Bx_{l}\left(t\right)1_{N}-x_{l}\left(t+1\right)1_{N}\\ = & f\left(t\right)1_{N}+\left(I_{N}-\beta \left(A_{\kappa (t)}+B\right)\right)\left(x\left(t\right)-x_{l}\left(t\right)1_{N}\right)\\ + & \left(I_{N}-\beta \left(A_{\kappa (t)}+B\right)\right)x_{l}\left(t\right)1_{N}+\beta Bx_{l}\left(t\right)1_{N}-x_{l}\left(t+1\right)1_{N}\\ = & \left(I_{N}-\beta \left(A_{\kappa (t)}+B\right)\right)e\left(t\right)-\beta A_{\kappa (t)}x_{l}\left(t\right)1_{N}\;. \end{array}$$

Since $A_{\kappa (t)}x_{l}\left(t\right)1_{N}=0$, we obtain

(13) $$e\left(t+1\right)=\left(I_{N}-\beta \left(A_{\kappa (t)}+B\right)\right)e\left(t\right)$$

and an error-switched system is as follows

(14) $$e\left(t+1\right)\in {\left\{\left(I_{N}-\beta \left(A_{\kappa }+B\right)\right)e\left(t\right)\right\}}_{\kappa \in I}\;.$$

**Remark** **3.** *Observe that transformation of system (11) to system (13) results in solving the stability problem of system (13) instead of the consensus problem of system (11).*

**Theorem** **3.** *Multi-agent system (11) under DoS attacks achieves a consensus with a leader, provided that matrices in the family ${\left\{I_{N}-\beta \left(A_{\kappa }+B\right)\right\}}_{\kappa \in I}$ are Schur stable.*

**Proof.**  Since, by assumption, all matrices in the family ${\left\{I_{N}-\beta \left(A_{\kappa }+B\right)\right\}}_{\kappa \in I}$ are symmetric and Schur stable, by use of Theorem 1, we get that error-switched system (14) is exponentially stable under arbitrary switching. Now, let us observe that exponential stability of switched system (14) implies that

$$\lim_{t\to \infty }|e_{i}\left(t\right)|=\lim_{t\to \infty }\left|x_{i}\left(t\right)-x_{l}\left(t\right)\right|=0\;,$$

which means that multi-agent system (11) under DoS attacks achieves a consensus with a leader. □

In order to give a simple condition on the coupling strength β that guarantees achieving a consensus with a leader, we set $spec\left(A_{\kappa }+B\right)=\left\{\gamma_{j}^{\kappa }:j=1,\ldots ,N\right\}$, κ∈I, and

(15) $$\gamma_{max}=\max_{j\in \{1,\ldots ,N\},\kappa \in I}\gamma_{j}^{\kappa }.$$

**Proposition** **2.** *If $\beta \in \left(0,\frac{2}{\gamma_{max}}\right)$, with $\gamma_{max}>0$ given by Formula (15), then multi-agent system (11) under DoS attacks achieves a consensus with a leader.*

**Proof.**  First let us observe that $spec\left({\left\{I_{N}-\beta \left(A_{\kappa }+B\right)\right\}}_{\kappa \in I}\right)=\left\{1-\beta \gamma_{j}^{\kappa }:j=1,\ldots ,N,\kappa \in I\right\}$. We show that $|1-\beta \gamma_{j}^{\kappa }|<1$ for all j=1,…,N and κ∈I. Since $\beta \in \left(0,\frac{2}{\gamma_{max}}\right)$, it follows that

$$\begin{array}{cc} & 0<\beta \gamma_{j}^{\kappa }<2\\ & -1<1-\beta \gamma_{j}^{\kappa }<1\\ & |1-\beta \gamma_{j}^{\kappa }|<1\;,\;\;\forall \;j=1,\ldots ,N\;,\kappa \in I\;. \end{array}$$

The latter means that all matrices from the family ${\left\{I_{N}-\beta \left(A_{\kappa }+B\right)\right\}}_{\kappa \in I}$ are Schur stable, and since they are also symmetric, by Theorem 1 we conclude that the error-switched system (14) is exponentially stable. It follows that

$$\lim_{t\to \infty }|e_{i}\left(t\right)|=\lim_{t\to \infty }\left|x_{i}\left(t\right)-x_{l}\left(t\right)\right|=0\;,$$

and the proof is complete. □

Now we illustrate the above results by numerical examples.

**Example** **1.** *Let us consider five agents with the dynamics described by (7) and two cases of leader’s dynamics, with constant ($x_{l}\left(t\right)\equiv 1$) and time-varying ($x_{l}\left(t+1\right)=x_{l}\left(t\right)+\sin\left(\frac{t}{3}\right)$) reference states. The initial conditions are X(0)=(1,0,1,1,0). We assume that there is information flow from a leader to the third and fourth agents, that is, B=diag{0,0,1,1,0}, and we calculate that $\gamma_{max}\approx 5,4$. In what follows, we apply the control law (10), and we examine the influence of the coupling strength β on the consensus with a leader under DoS attacks in the three cases. As the first one, we consider the system working without any interference and the matrix of the system of the form:*

$$\left[\begin{array}{ccccc} 4 & -1 & -1 & -1 & -1\\ -1 & 3 & 0\; & -1 & -1\\ -1 & 0 & 3 & 0 & -1\\ -1 & -1 & 0 & 4 & -1\\ -1 & -1 & -1 & -1 & 4 \end{array}\right]$$

*The second and the third cases are considered when DoS attacks take place and the matrices describing the attacked channels are the following:*

$$\left[\begin{array}{ccccc} 2 & 0 & 0 & -1 & -1\\ 0 & 2 & 0 & -1\; & -1\\ 0 & 0 & 1 & 0 & 0\\ -1 & -1 & 0 & 4 & -1\\ -1 & -1 & 0 & -1 & 3 \end{array}\right]$$

*and*

$$\left[\begin{array}{ccccc} 2 & -1 & -1 & 0 & 0\\ -1 & 1 & 0 & 0\; & 0\\ -1 & 0 & 3 & 0 & -1\\ 0 & 0 & 0 & 1 & 0\\ 0 & 0 & -1 & 0 & 1 \end{array}\right]\;.$$

**Figure 1:**
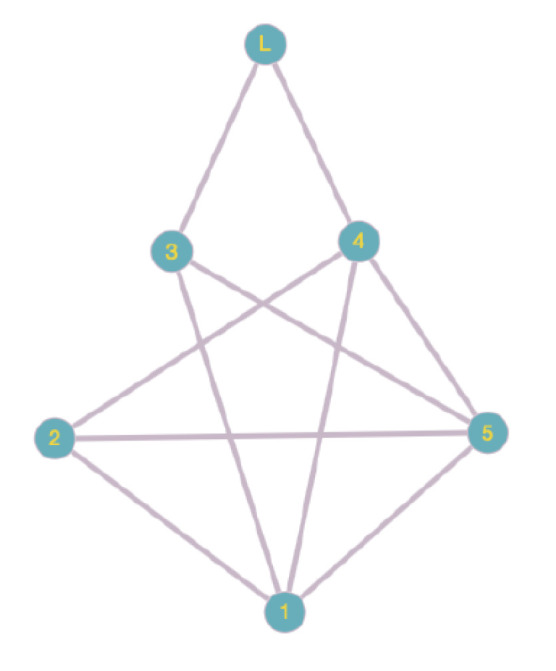
The interaction topology of the system without DoS attacks.

**Figure 2:**
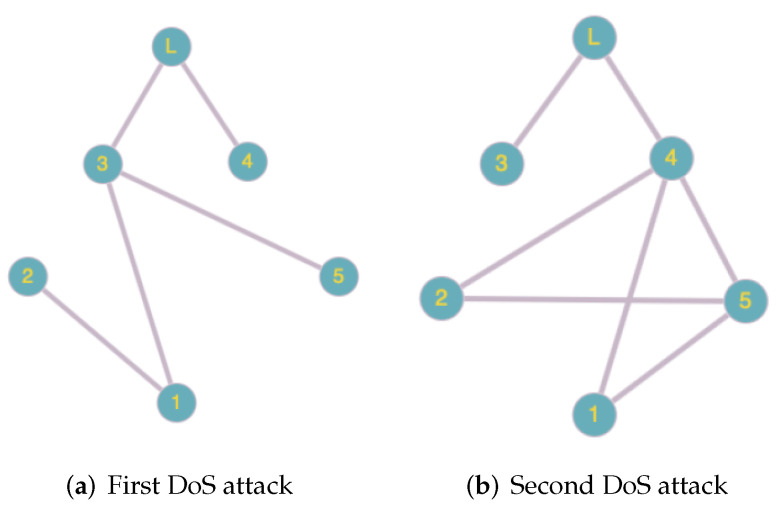
The interaction topologies of the systems with two DoS attacks.

*The interaction topologies for every case are presented in Figure 1 without DoS attacks and in Figure 2 with two consecutive attacks, respectively.*

**Constant reference state**

**Figure 3:**
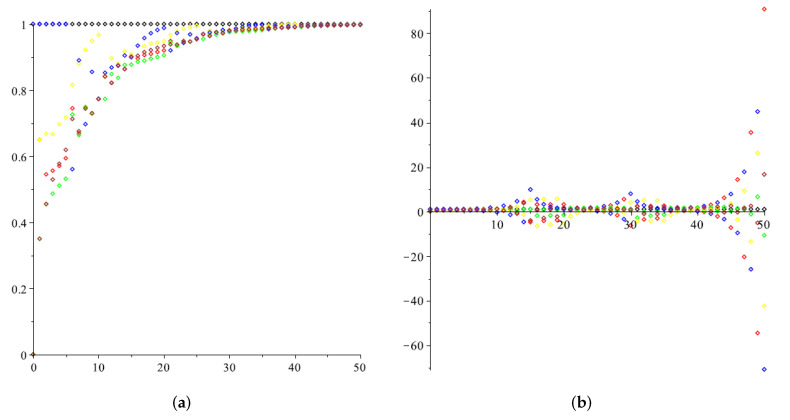
The multi-agent system with a constant reference state. (**a**) β= 0.35; (**b**) β= 0.5.

*Figure 3 illustrates the case when $x_{l}\left(t\right)\equiv 1$. In the first simulation, we choose β= 0.35 (Figure 3a), which fulfils the constraints of Proposition 2, and in the second β= 0.5 (Figure 3b), which does not. It is apparent that the consensus with a leader is achieved in the first case under the proposed controller.*

**Time-varying reference state**

**Figure 4:**
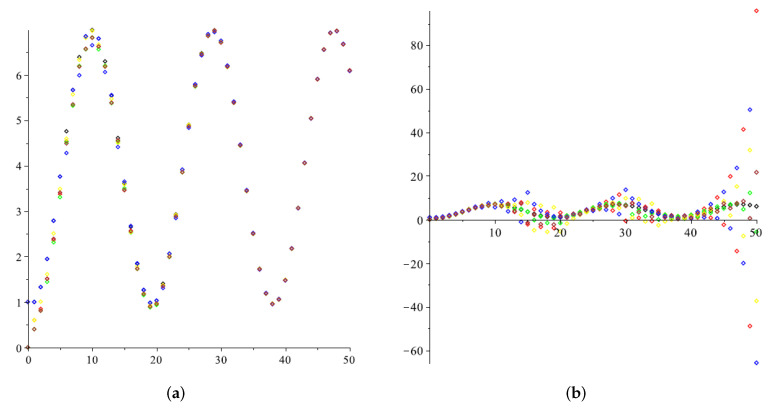
The multi-agent system with a time-varying reference state. (**a**) β= 0.35; (**b**) β= 0.5.

*Figure 4 shows the situation when $f\left(t\right)=\sin\left(\frac{t}{3}\right)$. As was already observed in the previous case if we choose β=0.35, then it fulfils the constraints of Proposition 2 and the consensus is achieved (Figure 4a), while for β=0.5 it does not (Figure 4b).*

## Data Availability

Not applicable.
